# Supplementary material for: Time-series transcriptome analysis identified differentially expressed genes in broiler chicken infected with mixed Eimeria species
Source: Front Genet. 2022 Aug 8;13:886781. doi: 10.3389/fgene.2022.886781 (PMC9393255; doi:10.3389/fgene.2022.886781)
Supplement: Supplementary file 2 [file DataSheet1.ZIP › 7dpi_KEGG.Gsea.1625115961950/KEGG_SMALL_CELL_LUNG_CANCER.html]

Details for gene set KEGG\_SMALL\_CELL\_LUNG\_CANCER[GSEA]

|  || Dataset | TMM\_7dpi\_gct\_format\_7dpi\_gct\_format.Class\_4dpi.cls #PC\_versus\_NC.Class\_4dpi.cls #PC\_versus\_NC\_repos |
| Phenotype | Class\_4dpi.cls#PC\_versus\_NC\_repos |
| Upregulated in class | 0 |
| GeneSet | KEGG\_SMALL\_CELL\_LUNG\_CANCER |
| Enrichment Score (ES) | -0.46513858 |
| Normalized Enrichment Score (NES) | -1.6105044 |
| Nominal p-value | 0.0025823112 |
| FDR q-value | 0.07936387 |
| FWER p-Value | 0.6346 |
Table: GSEA Results Summary

  

Fig 1: Enrichment plot: KEGG\_SMALL\_CELL\_LUNG\_CANCER      
 Profile of the Running ES Score & Positions of GeneSet Members on the Rank Ordered List

  

| SYMBOL | TITLE | RANK IN GENE LIST | RANK METRIC SCORE | RUNNING ES | CORE ENRICHMENT || 1 | CYCS | na | 475 | 0.763 | -0.0205 | No |
| 2 | BCL2L1 | na | 568 | 0.700 | -0.0106 | No |
| 3 | NOS2 | na | 593 | 0.688 | 0.0048 | No |
| 4 | MAX | na | 1375 | 0.411 | -0.0503 | No |
| 5 | LAMB4 | na | 1746 | 0.321 | -0.0732 | No |
| 6 | ITGA3 | na | 2012 | 0.270 | -0.0885 | No |
| 7 | CASP9 | na | 2022 | 0.268 | -0.0825 | No |
| 8 | BCL2 | na | 2059 | 0.261 | -0.0790 | No |
| 9 | TRAF4 | na | 2490 | 0.193 | -0.1101 | No |
| 10 | NFKBIA | na | 2532 | 0.187 | -0.1088 | No |
| 11 | RARB | na | 2792 | 0.151 | -0.1267 | No |
| 12 | ITGA2B | na | 2940 | 0.134 | -0.1356 | No |
| 13 | LAMB2 | na | 3242 | 0.094 | -0.1585 | No |
| 14 | CKS1B | na | 3323 | 0.084 | -0.1631 | No |
| 15 | CDK2 | na | 3911 | 0.015 | -0.2119 | No |
| 16 | BIRC3 | na | 4236 | -0.023 | -0.2384 | No |
| 17 | LAMA4 | na | 4435 | -0.043 | -0.2539 | No |
| 18 | TRAF1 | na | 5114 | -0.114 | -0.3078 | No |
| 19 | CCNE2 | na | 5260 | -0.127 | -0.3167 | No |
| 20 | LAMB1 | na | 5338 | -0.134 | -0.3198 | No |
| 21 | FHIT | na | 5463 | -0.145 | -0.3265 | No |
| 22 | CDKN1B | na | 5564 | -0.155 | -0.3310 | No |
| 23 | RELA | na | 6283 | -0.233 | -0.3852 | No |
| 24 | MYC | na | 6951 | -0.302 | -0.4335 | No |
| 25 | PTGS2 | na | 6986 | -0.306 | -0.4286 | No |
| 26 | CDKN2B | na | 7023 | -0.311 | -0.4238 | No |
| 27 | AKT3 | na | 7033 | -0.312 | -0.4166 | No |
| 28 | E2F1 | na | 7190 | -0.328 | -0.4214 | No |
| 29 | ITGB1 | na | 7209 | -0.331 | -0.4146 | No |
| 30 | RXRA | na | 7364 | -0.348 | -0.4187 | No |
| 31 | PIAS4 | na | 7394 | -0.351 | -0.4123 | No |
| 32 | CDK6 | na | 7686 | -0.382 | -0.4270 | No |
| 33 | CCNE1 | na | 7866 | -0.403 | -0.4318 | No |
| 34 | RB1 | na | 8176 | -0.443 | -0.4465 | No |
| 35 | AKT1 | na | 8399 | -0.472 | -0.4532 | Yes |
| 36 | IKBKB | na | 8465 | -0.479 | -0.4466 | Yes |
| 37 | PIK3CG | na | 8478 | -0.481 | -0.4355 | Yes |
| 38 | PTEN | na | 8620 | -0.503 | -0.4346 | Yes |
| 39 | APAF1 | na | 8687 | -0.512 | -0.4272 | Yes |
| 40 | FN1 | na | 8826 | -0.526 | -0.4255 | Yes |
| 41 | LAMC1 | na | 8856 | -0.531 | -0.4145 | Yes |
| 42 | PTK2 | na | 9133 | -0.570 | -0.4233 | Yes |
| 43 | NFKB1 | na | 9346 | -0.606 | -0.4257 | Yes |
| 44 | SKP2 | na | 9511 | -0.633 | -0.4235 | Yes |
| 45 | CHUK | na | 9554 | -0.639 | -0.4109 | Yes |
| 46 | ITGAV | na | 9564 | -0.640 | -0.3955 | Yes |
| 47 | PIK3R2 | na | 9597 | -0.645 | -0.3819 | Yes |
| 48 | COL4A1 | na | 9679 | -0.657 | -0.3721 | Yes |
| 49 | COL4A2 | na | 9798 | -0.674 | -0.3650 | Yes |
| 50 | E2F3 | na | 10031 | -0.717 | -0.3663 | Yes |
| 51 | TRAF2 | na | 10231 | -0.755 | -0.3640 | Yes |
| 52 | PIK3CB | na | 10335 | -0.777 | -0.3530 | Yes |
| 53 | COL4A4 | na | 10397 | -0.790 | -0.3382 | Yes |
| 54 | CCND1 | na | 10432 | -0.796 | -0.3209 | Yes |
| 55 | ITGA6 | na | 10612 | -0.836 | -0.3148 | Yes |
| 56 | PIK3CD | na | 10707 | -0.855 | -0.3011 | Yes |
| 57 | PIK3CA | na | 10920 | -0.912 | -0.2959 | Yes |
| 58 | PIK3R5 | na | 10941 | -0.918 | -0.2744 | Yes |
| 59 | TRAF5 | na | 10958 | -0.925 | -0.2524 | Yes |
| 60 | PIK3R3 | na | 11045 | -0.949 | -0.2356 | Yes |
| 61 | PIAS2 | na | 11080 | -0.959 | -0.2143 | Yes |
| 62 | TRAF6 | na | 11092 | -0.962 | -0.1909 | Yes |
| 63 | LAMA3 | na | 11104 | -0.965 | -0.1675 | Yes |
| 64 | LAMA1 | na | 11251 | -1.018 | -0.1540 | Yes |
| 65 | LAMA5 | na | 11261 | -1.022 | -0.1290 | Yes |
| 66 | PIK3R1 | na | 11265 | -1.023 | -0.1035 | Yes |
| 67 | ITGA2 | na | 11325 | -1.048 | -0.0820 | Yes |
| 68 | LAMB3 | na | 11340 | -1.052 | -0.0566 | Yes |
| 69 | TRAF3 | na | 11346 | -1.055 | -0.0304 | Yes |
| 70 | PIAS1 | na | 11577 | -1.157 | -0.0205 | Yes |
| 71 | RXRG | na | 12003 | -2.245 | 0.0006 | Yes |
Table: GSEA details [plain text format]

  

Fig 2: KEGG\_SMALL\_CELL\_LUNG\_CANCER      
 Blue-Pink O' Gram in the Space of the Analyzed GeneSet

  

Fig 3: KEGG\_SMALL\_CELL\_LUNG\_CANCER: Random ES distribution      
 Gene set null distribution of ES for **KEGG\_SMALL\_CELL\_LUNG\_CANCER**
